# Using Educational Comics to Increase Dementia Awareness and Promote Brain Health among Hispanic Women and Family Caregivers on the US/Mexico Border

**DOI:** 10.1002/alz70858_097851

**Published:** 2025-12-24

**Authors:** Sarah Y Jimenez, Christy Blanco, Clarissa S. Waletzko, Jacob Martinez, Maria Isela Maier

**Affiliations:** ^1^ University of Texas El Paso, El Paso, TX, USA; ^2^ Gayle Greve Hunt School of Nursing Texas Tech University Health Sciences Center El Paso, El Paso, TX, USA; ^3^ University of Texas at El Paso, El Paso, TX, USA

## Abstract

**Background:**

Alzheimer's disease and other dementias threatens the health and well‐being of older Latino adults and their caregivers. Latinos are affected at one and one‐half times the rate of White Caucasians. Two‐thirds of all dementia family caregivers are women. Dementia caregivers have higher‐than‐average rates of anxiety and depression. For this reason, educational workshops were developed to promote brain health among Latinas and to teach family caregivers stress‐reducing activities, including journaling and doodling. This study aimed to determine baseline dementia and brain health knowledge among Latinas and dementia caregivers, to assess the perceived stress levels of caregivers, and to evaluate if Latinos perceived educational comics as a useful health literacy tool.

**Method:**

A series of bilingual one‐hour journaling and doodling workshops for family caregivers and one‐hour brain health workshops for Latinas were conducted throughout El Paso County, Texas. Educational comic booklets illustrating brain health strategies and caregiver self‐care activities were presented. In this mixed‐methods pilot study, mean scores were obtained on the Dementia Knowledge Assessment Scale and Perceived Stress Scale among participants. A thematic analysis was conducted on data gathered through focus groups.

**Result:**

Dementia knowledge among Latina women and Latino dementia caregivers, and stress levels among the Latino dementia caregivers (Cohort 1‐Latino caregivers, *n* = 43; Cohort 2‐Latina women, *n* = 114) were assessed. Baseline Dementia Knowledge Assessment Survey (DKAS) scores revealed knowledge gaps in both cohorts (Cohort 1: 25.05 ± 7.66; Cohort 2: 18.39 ± 9.57/50 points), while Perceived Stress Scale (PSS‐10) scores in Cohort 1 indicated moderate stress (18.48 ± 5.37). Qualitative analysis revealed themes of enhanced relatability to comic storylines and characters and perceived enhanced understanding of brain health and dementia caregiving concepts.

**Conclusion:**

Educational comics can be efficacious as a health literacy tool for this population. This study supports further investigation into using educational comics to educate Latinos about improving brain health and expanding knowledge about Alzheimer's disease and other dementias to minimize the risk of disease development. This knowledge will support brain health practices and help those who live with Alzheimer's disease and other dementias develop stress‐management strategies that enhance quality of life as the disease progresses.

